# A Rare Encounter With Garre’s Osteomyelitis of the Proximal Tibia: A Case Report

**DOI:** 10.7759/cureus.62588

**Published:** 2024-06-18

**Authors:** Prashanth Balusani, Sandeep Shrivastava, Aditya Pundkar, Swapnil V Date

**Affiliations:** 1 Orthopaedics and Traumatology, Jawaharlal Nehru Medical College, Datta Meghe Institute of Medical Science, Wardha, IND; 2 Orthopaedic Surgery, Jawaharlal Nehru Medical College, Datta Meghe Institute of Medical Science, Wardha, IND

**Keywords:** non-suppurative osteomyelitis, multidisciplinary approach, tibial osteomyelitis, proliferative periosteal reaction, garre’s osteomyelitis, sclerosing osteomyelitis

## Abstract

Garre’s osteomyelitis, a rare form of chronic osteomyelitis, primarily affects the metaphyseal regions of long bones. This is frequently noted as an orthodontogenic infection in children and young adults. Dental infections are common underlying etiologies associated with Garre’s osteomyelitis. This case of a 47-year-old female describes a rare clinical presentation of proximal tibial-localized Garre’s osteomyelitis. The case highlights the diagnostic challenge of Garre’s osteomyelitis due to the age at presentation and its management, necessitating a multidisciplinary approach. The patient had a good prognostic outcome, attributable to the precision of the diagnostic modalities and the persistence of the treatment plans available at our tertiary care center. This study clarifies the complex nature of proximal tibia osteomyelitis, highlighting the need for accuracy and persistence in treating this uncommon and difficult orthopedic ailment when presented to individuals in the fourth decade of their lives.

## Introduction

Garre’s osteomyelitis is a distinctive type of bone infection characterized by non-suppurative and sclerosing features, leading to bone distension through cortical thickening, which was ﬁrst described by Carl Garre in 1893 [1]. This condition is characterized by a localized and tender segment of the affected bone. Typically, the mandible and metaphyses of long bones, particularly in pediatric patients, are the common sites of involvement [2,3]. Involvement of the diaphysis is infrequent and can imitate other conditions, necessitating careful exclusion using clinical expertise and comprehensive diagnostic evaluation. The diagnostic challenge is further compounded by the fact that bacterial cultures often yield negative results, adding to the complexity of the diagnosis [4]. Microorganisms such as *Streptococci sp.*, *Staphylococcus aureus*, *Staphylococcus pyogenes*, *Staphylococcus albus*, and other mixed strains are noted [2]. Increased levels of acute-phase inflammatory markers, such as C-reactive protein (CRP) and erythrocyte sedimentation rate (ESR), are also conceivable. Radiographic findings of this condition typically reveal cortical thickening and the absence of a visible medullary canal [5,6]. In cases of Garre’s osteomyelitis, there is no macroscopic suppurative lithic region, but histological studies have revealed microabscesses and microsequesters [7]. The primary objective of treatment is to alleviate symptoms. Though analgesics may provide relief for some patients, others might require antibiotic therapy. Surgical intervention is required in cases of inadequate disease control. Options for surgery include the removal of the chronic osteomyelitis-affected area to expose the medullary canal and debride the damaged bone [8-10]. This case report of a 47-year-old female highlights the importance of timely intervention and interdisciplinary teamwork regarding diagnosis and management. This is a rare presentation of Garre's osteomyelitis affecting the proximal tibia, with favorable outcomes achieved, and can provide a good insight to healthcare professionals.

## Case presentation

**Figure 1:**
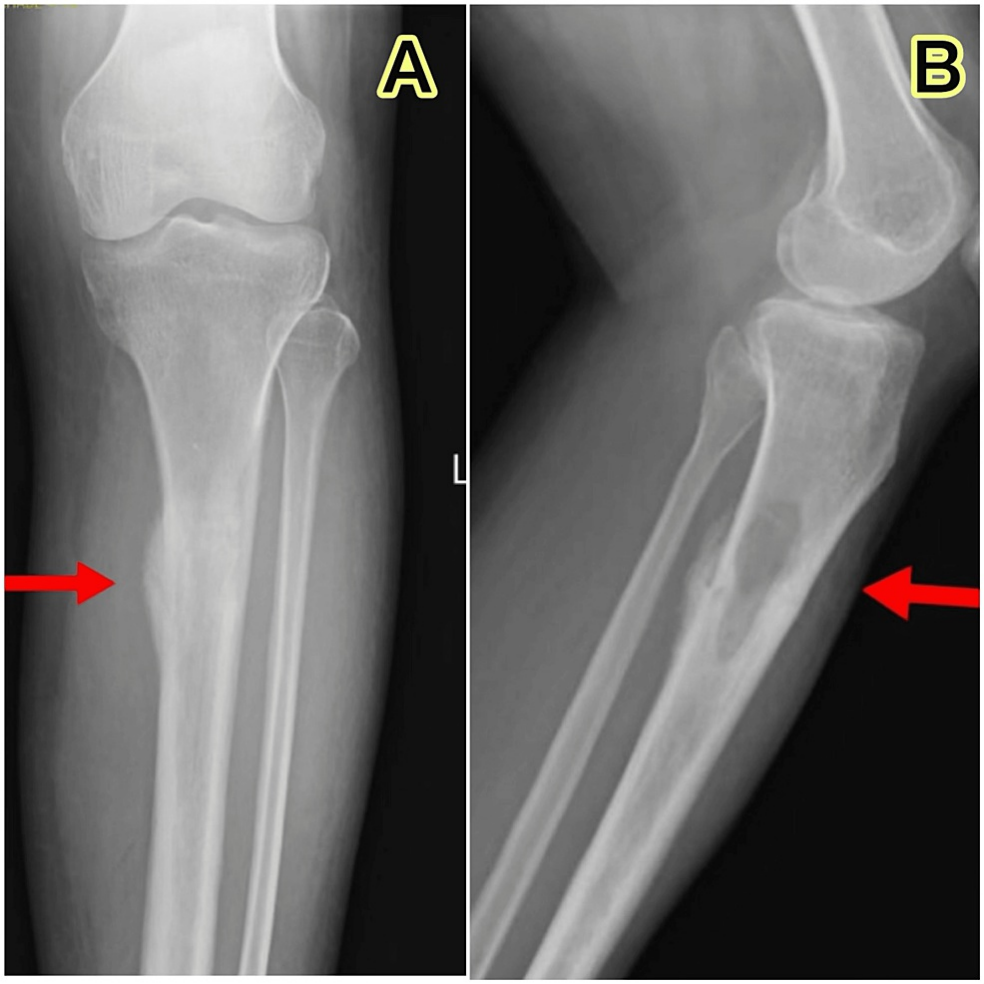
Radiographic image of left tibia and fibula Anterior-posterior view (A) and lateral view (B) show cortical thickening of the proximal one-third shaft of the left tibia; arrows indicate the thickening.

A 47-year-old female of South-Asian descent, working as a tailor, presented to the orthopedics department with a major complaint of pain in her left leg for six months. There were no notable prior experiences like nausea, vomiting, fever, trauma, or falls. There were no aberrant gaits, focal neurological deficits, localized edema, skin abnormalities, or pain found during a physical examination. White blood cell count, hemoglobin, and platelet count were within normal limits, despite the presence of increased inflammatory markers, most notably CRP and ESR (63 mg/L and 52 mm/h, respectively). The rest of the blood test parameters were within the normal range. Radiographic imaging of the left leg revealed a thickened cortex near the tibia’s shaft, with the fibula noted to be in good condition (Figure 1).

**Figure 2:**
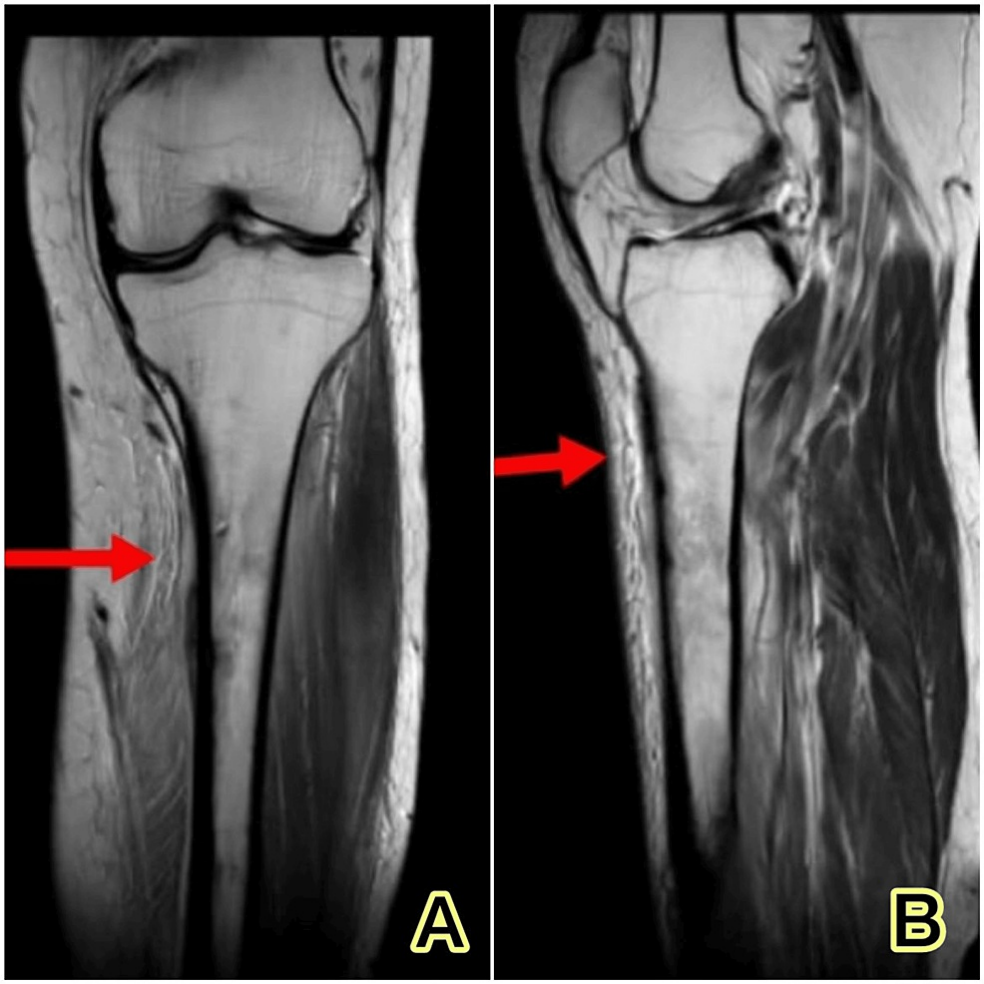
An MRI of the left tibia and fibula shows sub-periosteal fluid collection and irregular erosion of the medial cortex of the proximal one-third shaft of the tibia, suggesting the possibility of sclerosing osteomyelitis. A: anteroposterior view; B: lateral view

An MRI was done, and the results indicated that sclerosing osteomyelitis may be present due to sub-periosteal fluid accumulation and uneven erosion of the medial cortex of the proximal third shaft of the tibia (Figure 2).

**Figure 3:**
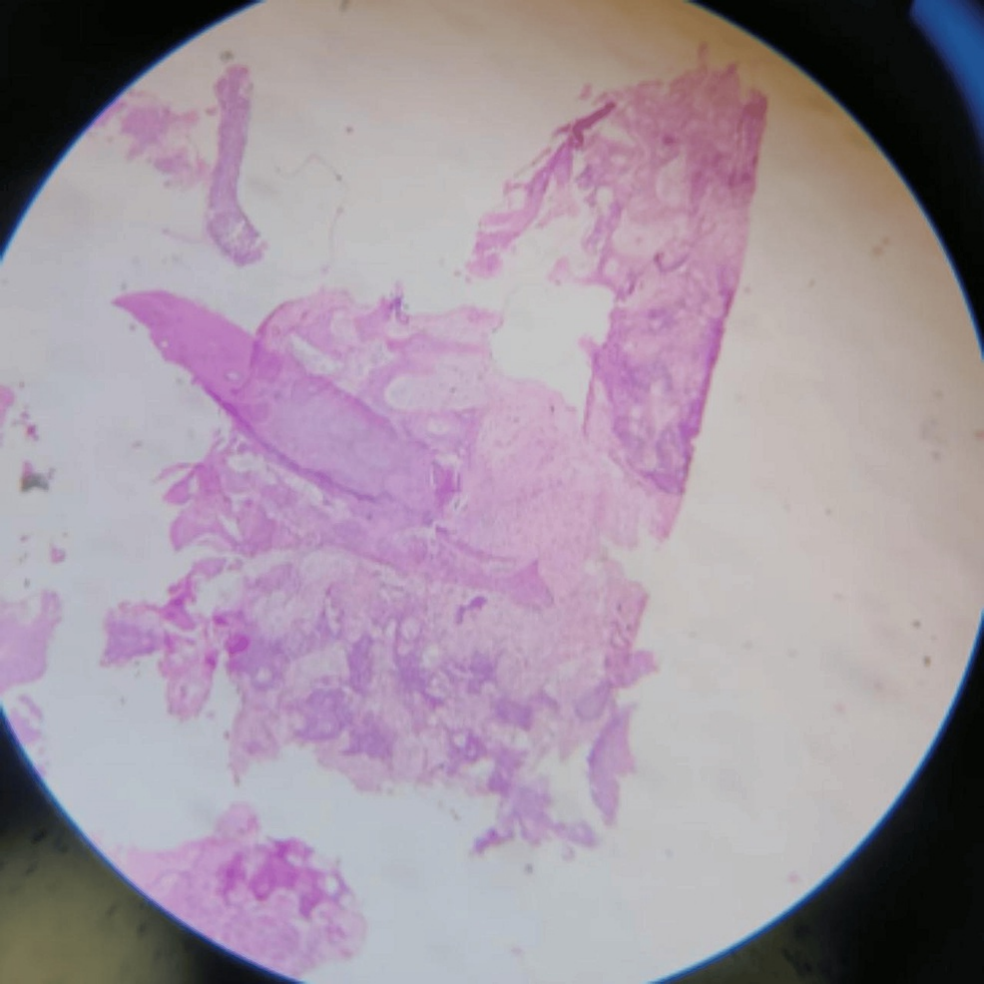
Hematoxylin-eosin staining of the specimen shows a mature bony cortex with indicators of chronic inflammation at 400x magnification

Following a core biopsy of the left proximal part of the tibia, histopathological analysis revealed chronic osteomyelitis as opposed to neoplastic disease, and it was characterized by sclerotic cortical bone fragments with fibrotic tissue and some inflammatory cell infiltrations composed of neutrophils, eosinophils, and a few lymphocytes in the marrow space (Figure 3).

The orthopedic team treated the patient during her hospital stay with a brief course of antibiotics which included injection of ceftriaxone and sulbactam 1.5 grams intravenous twice daily for five days, followed by oral antibiotics (tab ofloxacin 600 mg twice a day (BD)) for three months with the advice of physiotherapy. The patient was followed up every month for three months. At the three-month follow-up, the patient was mobilized without a walker, and the pain subsided. The knee's range of motion (ROM) was full without pain.

## Discussion

Garre’s osteomyelitis is an inflammation of the bones, also referred to as Garré’s sclerosing osteomyelitis, often due to its onion-skin-like appearance [11]. It is common in young adults and children but is rarely observed in individuals >40 years of age or later. Though there are cases reported for the same [2,6-8]. The clinical presentations associated with Garre's osteomyelitis are bone inflammation or bone swelling, dental infections noted with facial asymmetry, asymptomatic swelling, or insidious pain [6,8,11,12]. Cases with femur and tibia infections have also been available in the published literature, usually of >25 years of age [11]. Garré’s results suggested that a peripheral bone reaction brought on by a little infection or irritant was the cause of the periosteum thickening in long bones, but the precise etiology of this illness is not yet clearly understood. Although microbial infections such as *Staphylococcus*, *Klebsiella*, and *Streptococcus *are majorly suspected [2,4]. Traditional cultures frequently produce negative findings, pointing to the presence of low-virulence pathogens or chronic infections despite therapy. Additional research should be considered, such as polymerase chain reaction (PCR), when standard culturing is unable to identify the causal culprit [10,13]. Differential diagnosis of tumorous conditions such as osteoid osteoma, Ewing’s sarcoma, osteosarcoma, and eosinophilic granuloma must be ruled out. Other differential diagnoses for multifocal sclerosing bone responses may be considered, including fibrous dysplasia, syphilis, and pustulosis palmoplantar. Thorough diagnostic and comprehensive assessments are necessary to distinguish between Garre’s osteomyelitis and various other illnesses [7,12,13].

Diagnosis can be carried out by radiological imaging such as X-ray, MRI, and CT imaging, along with supportive blood investigations such as CRP, ESR, and a complete blood count [10,12]. There are no ideal treatment guidelines. Nonsteroidal anti-inflammatory medicines (NSAIDs), analgesics, and antibiotics (oral/intravenous) can be used initially in the form of a broad range, further modifying to a specified drug and dose based on culture results, with some cases requiring long-term therapy of six to eight weeks [10]. Surgical intervention might be considered in cases of poor disease control, followed by a long-term antibiotic regime to avoid recurrence. Fenestration and curettage at the afflicted spot are reported to be used in surgical management [4,8,10]. Spiral CT has been reported as the most accurate imaging modality in research involving 121 patients in the diagnosis of limb osteomyelitis. It achieved a sensitivity of 99.1%, a specificity of 80%, and an accuracy of 96.7%. In contrast, radiography had 81.8% accuracy, 84.9% sensitivity, and 60% specificity. Exacerbations had periosteal responses and bone growth, which looked like the layers of an onion. The thickening and sclerosis of the afflicted bone, which are hallmarks of Garre's osteomyelitis, are supported by these radiographic findings [8,10,14].

This case had proximal tibial Garre’s osteomyelitis. In addition to clinical correlation and the elimination of various tumorous and sclerotic bone disorders, the diagnosis was made based on the distinctive radiographic findings of cortical thickening and loss of the medullary canal. As Garre’s osteomyelitis commonly affects the mandible and long bone metaphysis, especially in juvenile patients, the rarity of diaphysis involvement contributes to the rarity of this case. Due to the chronic symptoms in this situation, surgical intervention was contemplated. The afflicted area was cleaned up as part of the fenestration and curettage procedures. Using this method was intended to lessen discomfort, reduce swelling, and accelerate recovery. Additionally, several other surgical procedures, including intramedullary reaming and intramedullary nailing, have been documented in the literature [13].

## Conclusions

This case study is meant to serve as a reminder of the significance of predicting underlying illness in refractory cases as well as the valuable contribution of sophisticated imaging technologies in supporting diagnostic and therapeutic choices. Overall, this case's positive outcome shows that Garre’s osteomyelitis can be treated successfully with focus, tenacity, and a multidisciplinary strategy. It underscores the significance of continuing care and emphasizes the value of customized treatment strategies.
